# Effects of Rare Earth Oxides on the Mechanical and Tribological Properties of Phenolic-Based Hybrid Nanocomposites

**DOI:** 10.3390/polym16010131

**Published:** 2023-12-30

**Authors:** Shenglian Wang, Shuang Chen, Jiachen Sun, Zimo Liu, Dingxiang He, Shaofeng Xu

**Affiliations:** 1 School of Intelligent Manufacturing and Automotive Engineering, Gannan University of Science and Technology, Ganzhou 341000, China; 9320100368@gnust.edu.cn; 2 College of Mechanical and Electrical Engineering, Jiangxi University of Science and Technology, Ganzhou 341000, China; 3 School of Resources and Architectural Engineering, Gannan University of Science and Technology, Ganzhou 341000, China

**Keywords:** phenolic-based hybrid nanocomposites, rare earth oxides, nano-silica, mechanical and tribological properties, orthogonal experimental design, fuzzy comprehensive evaluation method, brake friction materials

## Abstract

The incorporation of rare earth oxides and nano-silica has been found to significantly enhance the mechanical and tribological characteristics of phenolic-based hybrid nanocomposites. In this work, the impact of these additives was investigated through single-factor experiments. The study revealed that cerium oxide and yttrium oxide were the primary factors influencing changes in the impact strength, shear strength, coefficient of friction, and wear rate. Additionally, the content of nano-silica exerted the most substantial influence on the hardness and compressive strength of the specimens. Furthermore, the material ratios of the phenolic-based hybrid nanocomposites were optimized using an orthogonal experimental design and a fuzzy comprehensive evaluation method. The optimal material ratio for these nanocomposites was determined to be 2% cerium oxide, 2.5% yttrium oxide, and 3% nano-silica, based on their mechanical, frictional, and wear properties. This research provides valuable insights for the development of new brake friction materials with low friction and high wear resistance and contributes to meeting the demand for polymer composites with superior mechanical performance in diverse applications.

## 1. Introduction

Brake friction pads, generally made in the shape of a flat cylinder, play a vital role in maintaining the safety and reliability of the wind turbine yaw system [1,2]. To fully utilize wind energy, the yaw system keeps the wind blades in the wind turbine on the windward side, which maximizes the power generation efficiency [3,4]. Therefore, the yaw system needs to be activated frequently, requiring friction materials with a low wear rate and high heat resistance. Meanwhile, the friction pads rub against the brake disk during the yaw, causing wear and tear, which reduces the performance and service life of the friction pads [5,6]. Hence, the development of high-quality brake friction pads has become an important goal in the field of friction materials and the wind power industry.

Polymer composites are widely used in daily life and industry due to their exceptional properties in mechanics, tribology, thermal, and electrical [7,8,9]. This type of material consists of three essential components: polymer matrix, fibers, and fillers. Fibers serve as the primary load carriers within the composite structure. Recently, natural fibers such as bamboo, cotton, hemp, wool, and silk have gained attention as potential reinforcement materials in polymer composites [10,11]. This is due to their widespread availability, renewable nature, low density, effective thermal insulation, mechanical properties, biodegradability, reduced tool wear, and cost-effectiveness [12,13]. Gürgen, S. et al. conducted a study on the wear characteristics of ultra-high molecular weight polyethylene (UHMWPE)/SiC composites at elevated temperatures [14]. Their findings indicate that the composites demonstrate an escalated rate of material degradation with increasing temperature within the frictional system. Shanmugam, S. et al. investigated the impact of graphene on the microhardness and wear characteristics of graphene hybrid (flax/E-glass/epoxy) fiber-reinforced polymer (HFRP) composites using pin-on-disk tests [15]. The inclusion of graphene in the HFRP composites resulted in notable improvements in the thermal stability, microhardness, and tribological performance compared to the HFRP composites, attributed to the formation of robust covalent bonds between the graphene and the epoxy matrix. In particular, polymer nanocomposites with hybrid fillers such as rare earth oxides and nano-particles are becoming increasingly significant due to their multifunctional effect [16,17,18]. Such materials can meet the highest requirements for both mechanical and tribological properties [19,20,21]. Phenolic-based nanocomposite brake friction pads are preferred due to their durability in dry friction and resistance in harsh environments [22,23].

Many studies have shown that the doping of rare earth elements can significantly improve the tribological properties of phenolic-based hybrid nanocomposites [19,22,24,25]. Yu et al. designed a kind of polyimide nanocomposite filled with different rare earth oxides based on carbon nanotubes and graphene oxide, finding that filling with cerium oxide can dramatically reduce the friction coefficient and wear rate [19]. Zheng et al. investigated the effect of rare earth lanthanum oxide on the tribological properties of phenolic-based composites under oil lubrication conditions [26]. It is shown that the bamboo fiber and matrix were tightly bonded after adding lanthanum oxide, resulting in a change in the wear form of the specimen to slight fatigue wear. At the same time, the effect of different processes of rare earth solutions on the tribological properties of phenolic-based brake materials was investigated, and the results showed that rare earth can improve the interfacial bonding between fibers and resins [27]. Pan et al. fabricated rare earth lanthanum oxide microparticles-reinforced polyimide composites with the aim of improving the tribological properties [24]. The experimental results indicated that the friction coefficient showed a significant decrease and the anti-wear property was enhanced dramatically after adding lanthanum oxide microparticles. Meanwhile, some studies [28,29,30,31] found that the mechanical properties of phenolic-based hybrid nanocomposites can be dramatically ameliorated by adding nano-silica to nanocomposites.

In this study, rare earth cerium oxide, yttrium oxide, and nano-silica were used as modified materials to fill nanocomposites. The effect of cerium oxide, yttrium oxide, and nano-silica on the mechanical properties as well as the tribological properties were investigated through the orthogonal experimental design and fuzzy comprehensive evaluation method [32,33,34,35,36]. By carefully calibrating the proportions of the modified materials based on scientific principles, it is possible to produce brake friction pads with enhanced performance, thereby minimizing the need for extensive testing and enhancing the efficiency of the development process. 

## 2. Materials and Methods

### 2.1. Experimental Materials

The material substrates mainly were phenolic resin (PF2123 binder) and aramid fiber (as the reinforcing fiber). The hybrid fillers used in this study consisted of various materials, including magnesium oxide (Mohs hardness is 6), copper powder (granularity is 200 mesh, melting point is 1083 °C, the density is 8.9 g/cm^3^, purity is 99.9%), barite (granularity is 200 mesh), graphite particles (granularity is 100 mesh, the carbon content is more than 95%, melting point is 3625 °C), clay and iron powder (granularity is 200 mesh) [37,38]. The rare earth oxides used in the experiment were cerium oxide (CeO_2_) and yttrium oxide (Y_2_O_3_). Their relevant properties are shown in Table 1.

**Figure 1:**
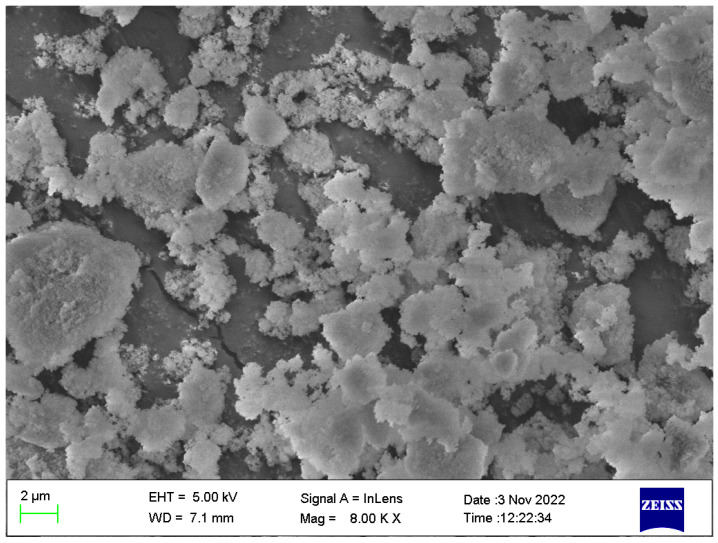
Microscopic morphology of nano-silica determined via scanning electron microscopy (SEM).

In this study, the hydrolysis method was used to prepare the nano-silica powder, and KH550 was used as the coupling agent [39,40]. The characteristics of nano-silica modified by the silane coupling agent KH550 are shown in Table 2, and the microscopic morphology is shown in Figure 1, which presents a fluffy spherical powder with some agglomerations and an average particle size of 30 nm.

### 2.2. Methods

In this study, phenolic-based nanocomposite friction pads with varying amounts of CeO_2_, Y_2_O_3,_ and nano-silica were fabricated using hot press molding technology to meet the performance requirements of wind-power yaw brake pads.

**Figure 2:**
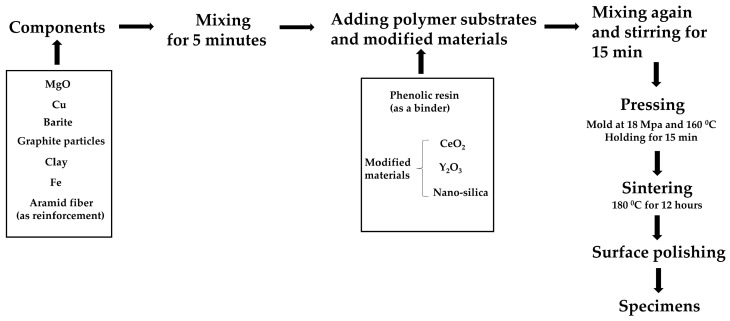
The sample preparation process.

Firstly, magnesium oxide, copper powder, barite, graphite particles, clay, and iron powder were mixed in the specified mass ratio (as shown in Table 3). Aramid fiber was added as a reinforcement at 3% of the mass of the mixed powder. The above mixture was placed in a dual-motion mixer and mixed for 5 min. Then, phenolic resin was added as a binder, and its addition was 24% of the mass of the mixed powder. Simultaneously, CeO_2_ powder, Y_2_O_3_ powder, and nano-silica powder were added as modified materials, the additive amount of which was 4%, 1%, and 1% of the mass of the mixed powder, respectively. Mixing again and stirring for 15 min. Subsequently, the stirred materials were placed in a 350-ton hot press for pressing and molding to obtain uniformly mixed molded materials. The working pressure, working time, and working temperature of the hot press were set at 18 MPa, 15 min, and 160 °C, respectively. The molded specimens were then put into an electric blast drying oven for heat treatment at a temperature of 180 °C for 12 h. Finally, the heat-treated phenolic-based nanocomposite friction pads were polished using a surface grinder. The sample preparation process is schematically shown in Figure 2.

**Figure 3:**
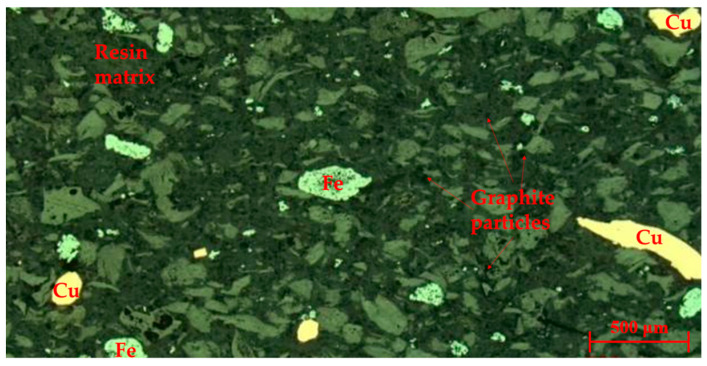
Microscopic morphology of the specimen.

The surface microscopic morphology of the specimen is shown in Figure 3. The gray area is the resin matrix, the bright white tissue is iron powder particles, the yellow tissue is copper powder, and the black region is graphite particles. In addition, the few white particles are magnesium oxide.

During the preceding time frame, a multitude of preliminary experiments was carried out to evaluate different hypotheses, resulting in the acquisition of valuable insights. In this study, the contents of cerium oxide, yttrium oxide, and nano-silica were used as the three factors in the mechanical and tribological performance tests, and four levels of each factor were set. An orthogonal test table was designed and obtained, as shown in Table 4. A total of 16 sets of specimens with different parameters in the orthogonal test table were selected, the concrete levels of which are listed in Appendix A.

The effects of the three factors on the hardness, impact strength, compressive strength, shear strength, friction coefficient, and wear rate of the phenolic-based nanocomposite friction materials were analyzed by employing the range analysis method to study the results of the orthogonal experimental design. Range analysis is a statistical method used to determine the factors’ sensitivity to the experimental result according to the orthogonal experiment [41,42]. Range is defined as the distance between the extreme values of the data. The greater the range is, the more sensitive the factor is. The material formulation with optimum performance was selected through the fuzzy comprehensive evaluation method. Fuzzy comprehensive evaluation is an application of fuzzy mathematics. It uses the principle of fuzzy transformation and the maximum membership degree, evaluating all the relevant factors to perform a comprehensive evaluation. This is an efficient evaluation method to evaluate objects that are affected by various factors.

In this study, the Vickers hardness was measured using the SHYCHVT-5Z image processing Vickers hardness tester produced by Huayin Testing Instrument Co., Ltd, Huayin, China. The impact strength was tested using the XJJ-5 impact tester produced by Chengde Testing Machine Co., Ltd, Chengde, China. The compressive and shear strength of the specimens were surveyed using the WAW-600KN universal hydraulic testing machine produced by Ruite Testing Machine Co., Ltd, Changshu, China. And the XD-MSM-type fixed speed friction tester (Chengde Testing Machine Co., Ltd, Chengde, China) was used to test the friction and wear performance of the specimens.

## 3. Results

### 3.1. Mechanical Properties

#### 3.1.1. Vickers Hardness and Impact Strength

**Figure 4:**
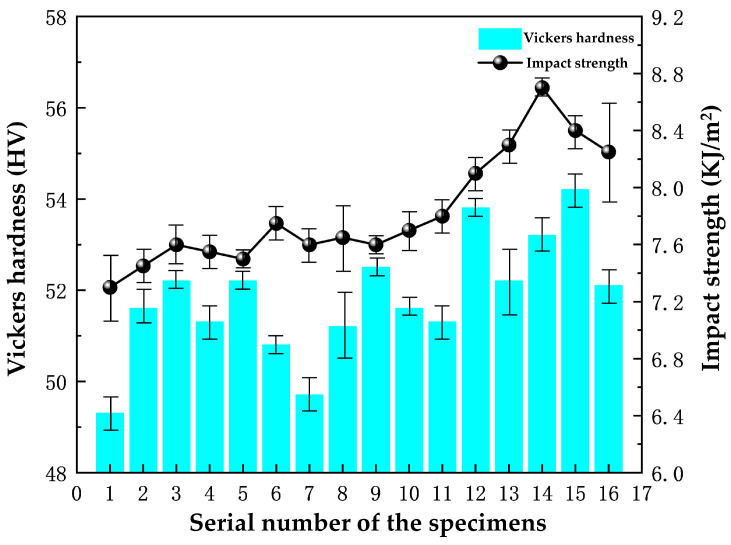
Vickers hardness and impact strength of the 16 sets of specimens.

The Vickers hardness and impact strength of the 16 sets of specimens with different parameters in the orthogonal test table are shown in Figure 4. Increasing the content of cerium oxide, yttrium oxide, and nano-silicon dioxide led to an overall increase in the Vickers hardness, ranging from 49 to 55 HV. Specimens 12 and 15 have a relatively high Vickers hardness, with specimen 15 having the highest value of 54.2 HV, while specimen 1 has the lowest at 49.3 HV. The error bars in Figure 4 show that the data fluctuate over a wide range, probably due to the fact that only three measurements were taken for each specimen.

The impact strength test results show that specimen 14 has the highest impact strength, measuring at 8.7 KJ/m^2^, while specimen 1 has the lowest impact strength with a value of 7.3 KJ/m^2^. However, all the specimens have impact strength values of at least 7 KJ/m^2^.

The range analysis of the Vickers hardness of the specimens is shown in Table 5. The K_1_ to K_4_ in Table 5 are the mean values at the same level for the different factors. Among the three factors involved in the experiment, nano-silica has the largest range value, followed by cerium oxide, and yttrium oxide has the smallest value. This indicates that nano-silica has the greatest influence on the Vickers hardness, while yttrium oxide has the least. Nano-silica particles are recognized for their exceptional hardness. In the process of manufacturing specimens through hot pressing, these particles are dispersed throughout the resin matrix diffusely. This distribution allows the particles to effectively fill any microcracks and pores in the materials. Therefore, nano-silica has an obvious effect on the enhancement of the hardness of the materials.

From the range analysis of the impact strength of the specimens in Table 6, it can be seen that the range of cerium oxide has the largest value, followed by yttrium oxide, and nano-silica has the smallest value. So, cerium oxide has the greatest effect on the impact strength. Cerium oxide and yttrium oxide have small differences in their range values, but both are much larger than those of nano-silica. The main reason is that rare earth elements can react with the hydroxyl group in the phenolic resin to produce stable compounds, which increases the interfacial bonding properties of phenolic resin. Thus, the fibers and the resin bond more tightly, which improves the specimens’ ability to withstand impact loads. At the same time, rare earth elements increase the surface area of the fibers, enhance the curing crosslinking effect of the fibers and fillers, and improve the impact toughness of the materials.

Based on a horizontal comparison of the mean impact strength values, it appears that the optimal levels of cerium oxide, yttrium oxide, and nano-silica would be 2.5%, 2.5%, and 2%, respectively, when aiming to improve the impact strength. This conclusion can be easily drawn after referring to both Table 4 and Table 6.

#### 3.1.2. Compressive Strength and Shear Strength

**Figure 5:**
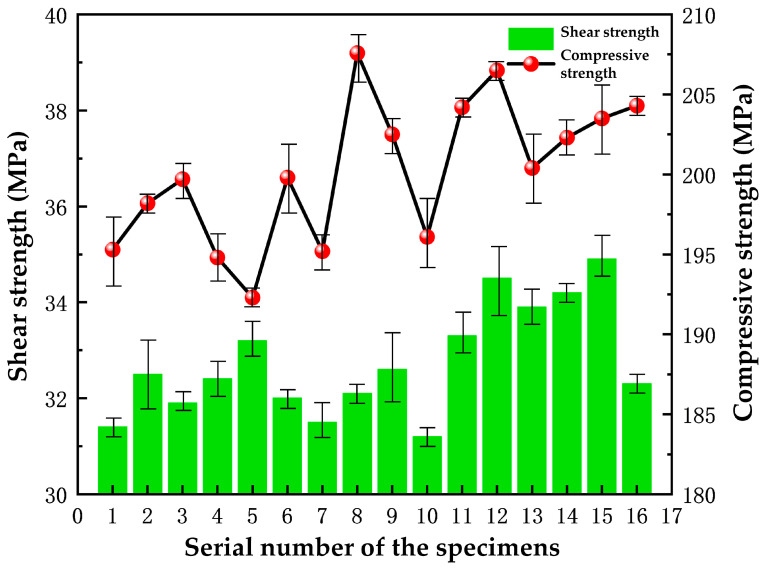
The compressive strength and shear strength of the 16 sets of specimens.

The compressive strength and shear strength of the 16 sets of specimens are shown in Figure 5. The compressive strength of the materials shows an increasing trend roughly with the increase in the cerium oxide content. It can be seen that the compressive strength values of specimens 8 and 12 are larger than the others. Specimen 5 has the smallest compressive strength value of 192.3 MPa. As can be seen from Figure 5, the fluctuation in the measurement errors for the shear strength is overall smaller when compared to the compressive strength, which may be brought about by the difference in the number of measurements and the accuracy of the instruments.

The three factors are ordered by the range values as follows: nano-silica > yttrium oxide > cerium oxide, as shown in Table 7. This means that nano-silica has the greatest impact on the compressive strength. The compressive strength of the specimen decreases when the nano-silica content is 5%. This may be due to the agglomeration phenomenon caused by the increased content of nano-silica. In other words, the nano-silica cannot be uniformly dispersed in the materials, which results in a reduction in the compressive strength.

Upon horizontal comparison of the mean values, it appears that the optimal levels of cerium oxide, yttrium oxide, and nano-silica would be 2.5%, 2.5%, and 4%, respectively, when using the compressive strength as an indicator.

Based on the analysis, the shear strength of the specimens generally increases as the content of the modified materials increases. Among all the specimens, the highest shear strength was exhibited by specimen 15, which has a value of 34.9 MPa. On the other hand, both specimen 1 and specimen 10 have lower shear strength than specimen 15. The shear strength of the remaining specimens, however, fluctuates in the relatively small range of 31–35 MPa.

Based on the range analysis of the shear strength presented in Table 8, it is evident that cerium oxide has the highest value among the three modified materials. Therefore, cerium oxide has the most significant impact on the shear strength of the specimens. Nano-silica has the second-highest range value, and yttrium oxide has the smallest range value, which has the smallest effect on the shear strength. The cerium element in cerium oxide, with high chemical activity, can act as a bridge between phenolic resin and aramid fiber, effectively improving the bond strength between the two materials while reducing the impact of shear on the nanocomposites.

Based on the in-depth analysis of the graphs and range analysis tables concerning the hardness, impact, compressive strength, as well as the shear strength of the specimens, it can be seen that the modified materials cause different degrees of enhancement of the mechanical properties of the specimens. Nano-silica has the greatest effect on the hardness and compressive strength of the specimens, while cerium oxide and yttrium oxide have a bigger effect on the impact strength and shear strength. The friction pads’ working effect depends not only on their mechanical properties but also on their friction and wear performance. Therefore, the study will focus on the tribological properties of the materials.

### 3.2. Tribological Properties

#### 3.2.1. Effect of Rare Earth Oxides and Nanoparticles on the Friction Coefficient during the Heating Process

**Figure 6:**
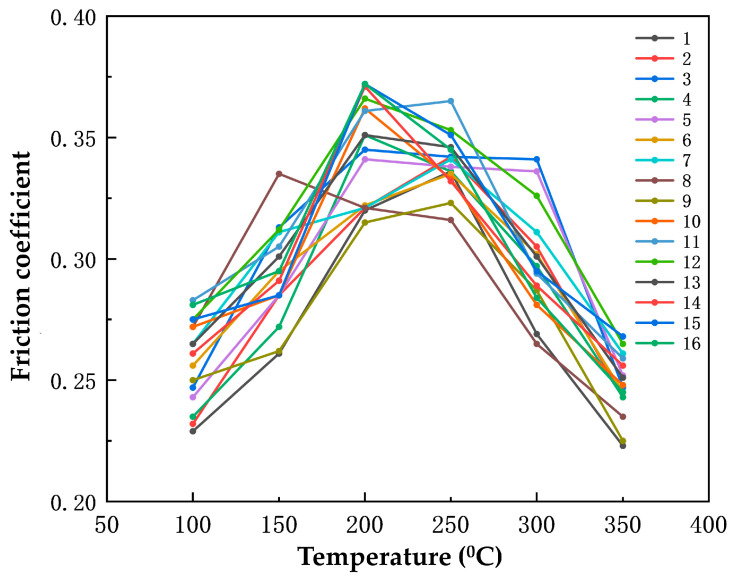
The friction coefficients of the 16 sets of specimens during the heating process.

Figure 6 shows the friction coefficients of the 16 sets of specimens during the heating process. The aim is to investigate the effect of cerium oxide, yttrium oxide, and nano-silica on the friction and wear performance of the phenolic-based hybrid nanocomposites using the range analysis method. With the temperature rise, the friction coefficients of the specimens generally increase initially and then decrease. When the temperature rises to 200~250 °C, the friction coefficients of most specimens reach their maximum and then begin to decline.

The decrease in the friction coefficients of the specimens is caused by the thermal decomposition of the phenolic resin inside them. This typically occurs when the temperature reaches 200–250 °C. As a result, the adhesion of the specimens decreases and the internal particle fillers start to shed, leading to a decrease in the friction coefficient.

The range analysis of the average friction coefficients of the specimens during the heating process is shown in Table 9. The range value of yttrium oxide is a maximum of 0.019, i.e., the influence of yttrium oxide on the friction coefficient is the maximum.

Combined with the graphs and range analysis tables, rare earth cerium oxide and yttrium oxide have a greater impact on the friction coefficient, and nano-silica has a smaller impact on the friction coefficient, during the heating process. Rare earth oxides possess exceptional interfacial characteristics and exhibit a unique electronic structure found in rare earth elements, which also display favorable chemical reactivity [43,44]. During high-temperature conditions, these oxides enhance the bonding of phenolic resin, promote material densification, and enhance the thermal decay resistance of the friction coefficient.

#### 3.2.2. Effect of Rare Earth Oxides and Nanoparticles on the Friction Coefficient during the Cooling Process

**Figure 7:**
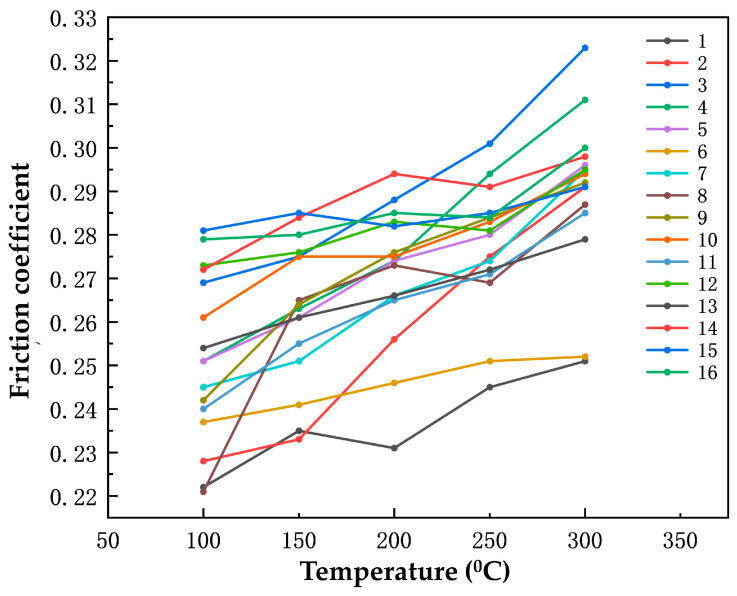
The friction coefficients of the 16 sets of specimens during the cooling process.

The graph in Figure 7 illustrates the relationship between the temperature and the friction coefficient of the specimens during the cooling process. It can be observed that as the temperature decreases, the friction coefficient gradually decreases. Specifically, when the temperature is reduced from 300 °C to 200 °C, the friction coefficients of the specimens decrease rapidly. However, during the subsequent stage of the temperature reduction from 200 °C to 100 °C, the decrease in the friction coefficient is less pronounced compared to the previous stage.

Based on the range analysis of the average friction coefficient during the cooling process presented in Table 10, it is evident that the range value of nano-silica is comparatively higher at 0.021, whereas the range values of cerium oxide and yttrium oxide are relatively lower at 0.019 and 0.016, respectively. This indicates that nano-silica has the most significant impact on the friction coefficient during the cooling process. The incorporation of nano-silica has the potential to alleviate the stress concentration on the aramid fibers and impede their failure.

#### 3.2.3. Effect of Rare Earth Oxides and Nanoparticles on the Wear Rate

**Figure 8:**
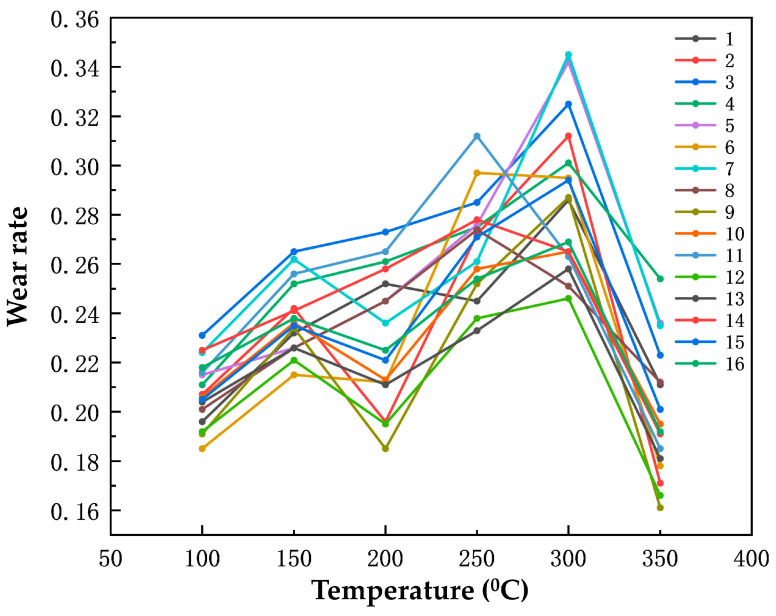
The wear rate varies with the temperature of the 16 sets of specimens.

The relationship between the wear rate of the specimens and the temperature is depicted in Figure 8. As the temperature increases, the wear rate initially rises and then declines. Between 100 °C and 250 °C, the wear rate increases gradually with the temperature elevation. In the range of 250 °C to 300 °C, the wear of the specimens intensifies, leading to a sharp rise in the wear rate. However, once the temperature reaches 300 °C, the wear rate of the specimen significantly decreases. At temperatures below 200 °C, the phenolic resin binder in the specimens exhibits strong bonding performance, resulting in a low wear rate. Conversely, when the temperature surpasses the thermal decomposition temperature of the phenolic resin, the resin begins to decompose, causing an increase in the wear rate of the specimens.

Based on the range analysis presented in Table 11, it is evident that the different concentrations of cerium oxide, yttrium oxide, and nano-silicon have varying effects on the wear rate of the specimens. Specifically, the wear rate is most significantly impacted by cerium oxide, with a maximum range of 0.022. Yttrium oxide follows closely, with a range of 0.021, while nano-silicon has the smallest impact on the wear rate, with a range of 0.08.

### 3.3. Fuzzy Comprehensive Evaluation

To achieve high-performance phenolic-based hybrid nanocomposites, this study employed the fuzzy comprehensive evaluation method to determine the weights of the relevant performance parameters of the specimens. Subsequently, the material ratio of the specimen with the highest score was selected as the optimal ratio of each factor.

#### 3.3.1. Fuzzy Evaluation of the Vickers Hardness and Impact Strength of Phenolic-Based Hybrid Nanocomposites

The hardness affects the actual contact area between the brake pad and the brake disc during the braking process. Excessive hardness reduces the actual contact area between the brake pad and the brake disc, leading to suboptimal braking performance and increased noise generation. Conversely, insufficient hardness results in excessive wear during braking. Appropriate impact strength can enhance the ability of the brake pad to resist damage from impact loads during operation, thereby improving the impact toughness. In the present experiment, the Vickers hardness of the phenolic-based hybrid nanocomposites ranged from 49 to 55 HV, with the fuzzy evaluation criteria for Vickers hardness set as shown in Table 12; the impact strength ranged from 7.3 to 8.7 KJ/m^2^, with the fuzzy evaluation criteria for impact strength set as shown in Table 13.

#### 3.3.2. Fuzzy Evaluation of the Compressive Strength and Shear Strength of Phenolic-Based Hybrid Nanocomposites

During the braking process, the friction pads on both sides of the brake disc are compressed against the brake disc under pressure to brake the wind turbine system. Elevating the compressive strength and shear strength values of the friction pads can enhance the overall material bonding strength, ensuring that the friction pads are less prone to fracture during braking. In this experiment, the compressive strength value of the friction pads ranged from 192 to 208 MPa, and the fuzzy evaluation criteria for compressive strength are presented in Table 14; the shear strength ranged from 31 to 35 MPa, and the fuzzy evaluation criteria are presented in Table 15.

#### 3.3.3. Fuzzy Evaluation of the Friction Coefficient of Phenolic-Based Hybrid Nanocomposites

The appropriate friction coefficient is one of the important performance indicators for friction pads. If the friction coefficient of the friction pad is too high, it is prone to excessive clamping between the friction pad and the brake disc during the braking process, leading to increased wear and consumption. Conversely, if the friction coefficient of the friction pad is too low, the braking system will experience prolonged braking time during emergency braking, resulting in the inability of the braking system to promptly align the wind blades with the wind direction, thereby reducing the efficiency of the wind energy conversion into electrical energy. In this experiment, the average friction coefficient of the friction pad ranged from 0.264 to 0.316, and the criteria for the fuzzy evaluation of the average friction coefficient are presented in Table 16.

#### 3.3.4. Fuzzy Evaluation of the Wear Rate of Phenolic-Based Hybrid Nanocomposites

The wear resistance of the yaw brake friction pad directly affects its service life, especially considering that wind turbines are often located in mountainous or coastal areas, making replacement difficult. Therefore, a higher wear resistance is required. The wear rate directly impacts the actual service life. In this experiment, the average wear rate of the friction pad ranged from 0.210 to 0.267, and the criteria for the fuzzy evaluation of the average wear rate are presented in Table 17.

To evaluate the friction materials, a comprehensive analysis of various properties, including hardness (H), impact strength (I), compressive strength (C), shear strength (V), coefficient of friction (μ), and wear rate (W), is necessary. To assign appropriate weights to these indicators, the friction coefficient is given a weight of 0.35, the wear rate is assigned a weight of 0.25, the Vickers hardness is given a weight of 0.1, the impact strength is assigned a weight of 0.1, the compressive strength is given a weight of 0.1, and the shear strength is assigned a weight of 0.1. The overall score (S) can be calculated using the Formula (1).

(1) S=0.35μ+0.25W +0.1H+0.1I+0.1C+0.1V

**Figure 9:**
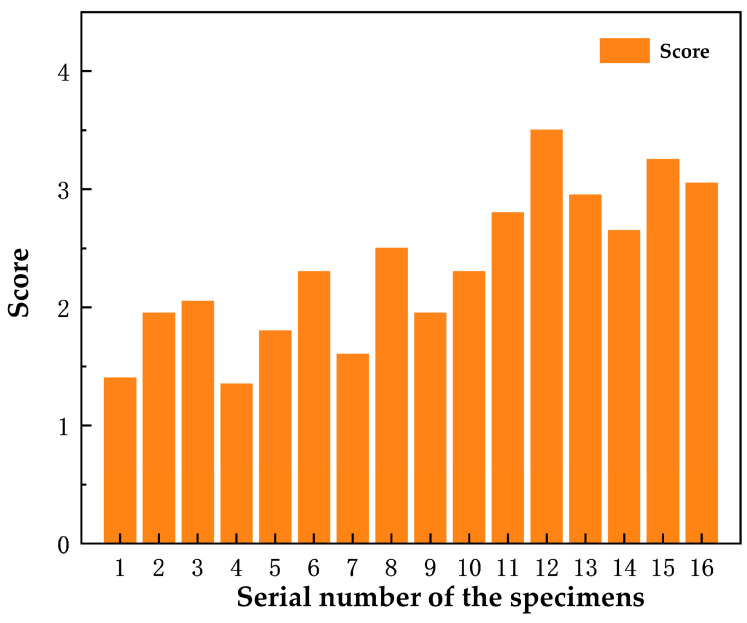
The scores of the 16 sets of specimens.

In this calculation as shown in Figure 9, specimens 13, 15, and 16 have higher comprehensive scores and better overall performance, while specimens 1 and 4 have worse comprehensive scores. Specimen 12 has the highest comprehensive score. The ratios of the three modified materials of specimen 12 are taken as the optimal solution, i.e., 2% cerium oxide, 2.5% yttrium oxide, and 3% nano-silica. To assess the reliability of these results, validation tests were conducted using these material ratios. The obtained results for the Vickers hardness, impact strength, compressive strength, shear strength, friction coefficient at 200 °C (during the heating process), and wear rate are 53.8 HV, 7.79 KJ/m^2^, 206.5 MPa, 34.5 MPa, 0.366, and 0.21, respectively. Comparing these validation results with the fuzzy predicted values, no significant difference is observed (*p* > 0.05). Therefore, it can be concluded that the optimized formulation parameters obtained through the fuzzy comprehensive evaluation method are accurate and reliable.

## 4. Conclusions

In this paper, 16 sets of specimens, which were tested using an orthogonal experimental design, were prepared according to different ratios of the three modified materials: cerium oxide, yttrium oxide, and nano-silica. The specimens were tested for relevant mechanical and tribological properties. The effects of the three modified materials on the performance of the phenolic-based hybrid nanocomposites were analyzed through range analysis of the test data, and the following conclusions were drawn:

(1) Cerium oxide, yttrium oxide, and nano-silica cause different levels of enhancement of the mechanical properties and tribological properties of the phenolic-based hybrid nanocomposites.
(2) When the levels of the three factors were changed, nano-silica had the greatest effect on the hardness and compressive strength. This mainly relies on the fact that nano-silica has a nano-size, which results in a large specific surface area and combines well with other component materials.
(3) Rare earth cerium oxide and yttrium oxide have a greater influence on the impact strength, shear strength, friction coefficient, and wear rate. This phenomenon can be attributed to the unique electron layer configuration of rare earth elements, which in turn enhances the interfacial bonding characteristics between the resin and fibers when rare earth oxides are employed.
(4) The material ratios of the phenolic-based hybrid nanocomposites were optimized through the orthogonal experimental design and fuzzy comprehensive evaluation method. The optimal formulation of the modified materials is 2% cerium oxide, 2.5% yttrium oxide, and 3% nano-silica.

This study demonstrated the beneficial impact of incorporating rare earth oxide additives on the mechanical and tribological characteristics of phenolic-based hybrid nanocomposites. It showed that incorporating rare earth oxides into nanocomposites results in heightened impact strength, shear strength, and wear resistance. Furthermore, the incorporation of nano-silica further enhances the compressive strength and hardness, attributed to the high aspect ratio of the nano-silica particles. These findings have potential implications for the advancement of brake friction materials with the desired low friction and high anti-wear properties, as well as for the production of polymer composites with enhanced mechanical performance in engineering applications.

## Figures and Tables

**Table 1:** Properties of CeO_2_ and Y_2_O_3_.

Material	Purity (%)	Granularity (Mesh)	Melting Point (°C)	Density (g/cm^3^)
CeO_2_	99.9	200	2397	7.13
Y_2_O_3_	99.9	200	2410	5.01

**Table 2:** Properties of nano-silica.

Material	Purity (%)	Particle Size (nm)	Melting Point (°C)	PH	Specific Surface Area (m^2^/g)
Nano-silica	99.8	30 ± 5	1750	5~7	150~300

**Table 3:** The mass fraction of the components before adding the polymer substrates and modified materials.

	Components						
	MgO	Cu	Barite	Graphite Particles	Clay	Fe	Aramid Fiber
**Mass** **fraction (%)**	10	15	22	15	20	15	3

**Table 4:** Factors and levels (mass fraction, %) of the orthogonal experimental design.

Factor	Levels (Mass Fraction, %)			
CeO_2_	1	1.5	2	2.5
Y_2_O_3_	1	1.5	2	2.5
Nano-silica	2	3	4	5

**Table 5:** Range analysis of the Vickers hardness of the specimens.

Factor	Mean Value (HV)				Range (HV)
	K_1_	K_2_	K_3_	K_4_	R = K_max_ − K_min_
CeO_2_	51.100	50.975	52.300	52.925	1.950
Y_2_O_3_	51.550	51.800	51.850	52.100	0.550
Nano-silica	50.875	52.950	52.275	51.200	2.075

**Table 6:** Range analysis of the impact strength of the specimens.

Factor	Mean Value (KJ/m^2^)				Range (KJ/m^2^)
	K_1_	K_2_	K_3_	K_4_	R = K_max_ − K_min_
CeO_2_	7.480	7.572	7.620	7.818	0.338
Y_2_O_3_	7.485	7.558	7.630	7.818	0.333
Nano-silica	7.675	7.595	7.555	7.665	0.120

**Table 7:** Range analysis of the compressive strength of the specimens.

Factor	Compressive Strength (MPa)				Range (MPa)
	K_1_	K_2_	K_3_	K_4_	R = K_max_ − K_min_
CeO_2_	197.000	198.725	202.325	202.625	5.625
Y_2_O_3_	197.625	199.100	200.650	203.300	5.675
Nano-silica	200.900	200.125	203.025	196.625	6.400

**Table 8:** Range analysis of the shear strength of the specimens.

Factor	Shear Strength (MPa)				Range (MPa)
	K_1_	K_2_	K_3_	K_4_	R = K_max_ − K_min_
CeO_2_	32.050	32.200	32.900	33.825	1.775
Y_2_O_3_	32.775	32.475	32.900	32.825	0.425
Nano-silica	32.250	33.775	32.700	32.250	1.525

**Table 9:** Range analysis of the friction coefficients during the heating process.

Factor	Friction Coefficients				Range
	K_1_	K_2_	K_3_	K_4_	R = K_max_ − K_min_
CeO_2_	0.289	0.296	0.300	0.304	0.015
Y_2_O_3_	0.288	0.294	0.307	0.300	0.019
Nano-silica	0.295	0.303	0.293	0.298	0.010

**Table 10:** Range analysis of the friction coefficients during the cooling process.

Factor	Friction Coefficients				Range
	K_1_	K_2_	K_3_	K_4_	R = K_max_ − K_min_
CeO_2_	0.266	0.262	0.274	0.281	0.019
Y_2_O_3_	0.262	0.267	0.276	0.278	0.016
Nano-silica	0.258	0.274	0.279	0.272	0.021

**Table 11:** Range analysis of the wear rate.

Factor	Wear Rate				Range
	K_1_	K_2_	K_3_	K_4_	R = K_max_ − K_min_
CeO_2_	0.249	0.246	0.227	0.233	0.022
Y_2_O_3_	0.233	0.234	0.254	0.234	0.021
Nano-silica	0.237	0.234	0.241	0.242	0.008

**Table 12:** Fuzzy evaluation criteria for the Vickers hardness.

Vickers Hardness (HV)	49~50.5	50.51~52	52.01~53.5	53.51~55
Score	1	2	3	4

**Table 13:** Fuzzy evaluation criteria for the impact strength.

Impact Strength (KJ/m^2^)	7.3~7.65	7.651~8.0	8.01~8.35	8.351~8.7
Score	1	2	3	4

**Table 14:** Fuzzy evaluation criteria for the compressive strength.

Compressive Strength (MPa)	192~196	196~200	200~204	204~208
Score	1	2	3	4

**Table 15:** Fuzzy evaluation criteria for the shear strength.

Shear Strength (MPa)	31~32	32~33	33~34	34~35
Score	1	2	3	4

**Table 16:** Fuzzy evaluation criteria for the friction coefficient.

Friction Coefficient	0.264~0.277	0.277~0.290	0.290~0.303	0.303~0.316
Score	1	2	3	4

**Table 17:** Fuzzy evaluation criteria for the wear rate.

Wear Rate	0.255~0.270	0.240~0.255	0.225~0.240	0.210~0.225
Score	1	2	3	4

## Data Availability

Data are contained within the article.

## Appendix A

**Table A1:** The concrete levels of the 16 sets of specimens in the orthogonal test table are listed below.

Factor	Levels (Mass Fraction, %)															
	Serial Number of the Specimens															
	1	2	3	4	5	6	7	8	9	10	11	12	13	14	15	16
CeO_2_	1	1	1	1	1.5	1.5	1.5	1.5	2	2	2	2	2.5	2.5	2.5	2.5
Y_2_O_3_	1	1.5	2	2.5	1	1.5	2	2.5	1	1.5	2	2.5	1	1.5	2	2.5
Nano-silica	2	3	4	5	3	2	5	4	4	5	2	3	5	4	3	2
